# Quantitative autofluorescence is increased in clinically unaffected fellow eyes from patients with posterior uveitis

**DOI:** 10.1038/s41598-025-90071-7

**Published:** 2025-02-26

**Authors:** Robert P. Finger, Julie Jungblut, Marie D. Just, Jan H. Terheyden, Frank G. Holz, Raffael Liegl, Thomas Ach, Maximilian W. M. Wintergerst

**Affiliations:** 1 Department of Ophthalmology, University Hospital Bonn, Venusberg Campus 1, 53127 Bonn, Germany; 2 Department of Ophthalmology, Medical Faculty Mannheim, Heidelberg University, 68167 Mannheim, Germany; 3 Augenzentrum Grischun, Chur, Switzerland

**Keywords:** Quantitative autofluorescence, Posterior uveitis, Endpoint, Imaging biomarker, Inflammatory eye diseases, Outcome measure, Uveal diseases, Retinal diseases, Acute inflammation, Chronic inflammation, Fluorescence imaging

## Abstract

The purpose of this prospective case-control study is to investigate differences in quantitative autofluorescence (qAF) in clinically affected and unaffected eyes of patients with inactive posterior uveitis compared to healthy, age-matched controls. Patients with posterior uveitis and healthy controls were imaged using fundus autofluorescence (488 nm excitation; Spectralis HRA + OCT; Heidelberg Engineering) to measure qAF values using the proprietary HEYEX software. Mean background qAF (excluding vessels and retinal lesions) across all segments (as previously defined by Delori et al.) and in the segment with the highest mean qAF value were compared between affected and unaffected eyes from patients with posterior uveitis, and healthy age-matched control eyes using the Kruskal-Wallis-test. A total of 83 eyes from 83 patients were included: 33 affected eyes (33 patients with uni-/bilateral posterior uveitis), 21 clinically unaffected eyes (21 patients with unilateral posterior uveitis), and 29 healthy, age-matched control eyes (29 patients). Mean qAF values were significantly higher (p-value < 0.0001) in both clinically affected (177.0 ± 83.8 qAF arbitrary units [qAF a.u.]) and unaffected (173.8 ± 56.4 qAF a.u.) eyes compared to healthy, age-matched controls (135.7 ± 41.8 qAF a.u.). Likewise, mean qAF in the segment with the highest mean qAF value was significantly higher (p-value: <0.01) in affected (243.2 ± 103.1 qAF a.u.) and unaffected eyes (227.1 ± 63.4 qAF a.u.) in comparison to controls (168.9 ± 48.5 qAF a.u.). In conclusion, both clinically affected and unaffected eyes from patients with posterior uveitis demonstrated increased fundus autofluorescence. The results of our study could indicate subclinical inflammation in currently inactive and (yet) unaffected eyes of posterior uveitis patients. This could be caused by accumulation of fluorophores or an increased metabolic activity generated by low-grade inflammation. As these changes may precede future inflammation in yet unaffected eyes, additional longitudinal studies including analysis of eyes with active disease are warranted.

## Introduction

Posterior uveitis is a group of diseases characterized by inflammation of the posterior segment, including choroid, retina and retinal vasculature. Despite its rare incidence and prevalence, uveitis causes 5–10% of worldwide blindness and vision impairment^1^ and affects predominantly people of working age^2–5^.

Immune privilege is a unique attribute of certain tissues and organs that allows them to tolerate foreign antigens without eliciting an immune response^6^. Immune privilege in the eye is crucial for maintaining ocular health as excessive immune activation can lead to irreversible damage and therefore loss of vision. Contrary to the earlier view that immune privilege is maintained by cell exclusion, recent studies have shown that active mechanisms, mainly performed by healthy retinal pigment epithelial (RPE) cells, suppress the response to antigens in the eye, thus limiting immune activation and inflammation within ocular tissue^7–9^.

Fundus autofluorescence (FAF) imaging is a non-invasive method that provides information about the topographic distribution of intrinsic fluorophores of the fundus that occur naturally as well as in association with disease^10–13^. Various previous studies have shown that FAF can assist in evaluating structural pathophysiological changes in posterior uveitis as well as help distinguish between different subtypes^14–16^. Durrani and Foster demonstrated that FAF is useful to identify early inflammation as well as past and future oxidative RPE damage in posterior uveitis^17^. Since the introduction of quantitative autofluorescence (qAF) by Delori et al. in 2011, it is possible to objectively quantify autofluorescence intensities among subjects and visits^18^.

Autofluorescence has been shown to be increased in chorioretinal inflammatory disorders^19–22^. It has also been demonstrated that FAF can help monitor and evaluate disease activity, prognosis and therapy^15,17,23–25^. However, to date, no studies have investigated unaffected eyes in posterior uveitis patients or background autofluorescence in clinically affected eyes outside the chorioretinal lesions. Therefore, we assessed autofluorescence quantitatively in (yet) clinically unaffected eyes in patients with posterior uveitis to investigate whether autofluorescence is altered due to potential sub-clinical inflammation.

## Materials and methods

### Subject recruitment

This prospective case-control study took place at the University Hospital Bonn, Department of Ophthalmology, Germany. Ethical approval was obtained from the ethics committee of the University of Bonn (approval ID 011/18) and all participants gave their informed consent before being included. The study was conducted in adherence to the Declaration of Helsinki. Patients with inactive posterior uveitis according to the diagnostic criteria of the Standardization of Uveitis Nomenclature (SUN) working group and established findings in clinical examination and imaging were included^26^. Clinical and demographic characteristics were obtained from the medical charts. Exclusion criteria were primary vasculitis, cataract reducing the image quality as assessed by examiner, or poor qAF image quality (see below).

### Image acquisition and analysis

The pupil was dilated with 0.5% tropicamide. All participants were imaged by one examiner, who was trained appropriately, with FAF using a confocal scanning laser ophthalmoscope with 488 nm excitation wavelength (Spectralis HRA + OCT; Heidelberg Engineering, Heidelberg, Germany). The qAF imaging was performed as previously described^27^. Concisely, the device simultaneously excites and records an internal reference during the qAF acquisition process, enabling comparison of FAF intensities intra- and inter-individually, as well as across follow-up examinations. Room lights were turned off, and the camera was positioned centered on the fovea by using the near-infrared reflectance mode and the internal fixation light. After switching to the qAF mode (488 nm excitation wavelength and 500 to 680 nm detection), focus and alignment were adjusted to obtain a maximum and uniform signal. For bleaching of the FAF signal, the photoreceptors were exposed to 488 nm excitation wavelength laser light for 20 to 30 s to bleach the photopigments and to ensure reliable qAF signal acquisition. At least three series of twelve successive images were recorded in high-speed mode and the camera was newly positioned after each image series. The recordings were performed using customized software developed by Heidelberg Engineering for recording qAF images (30° field of view, 768 × 768 pixels). If necessary, low-quality frames (e.g., lacking focus, unstable fixation, eyelashes or eye lid interference, image not centered on the fovea) were deleted manually. The remaining frames were averaged into one mean qAF image by the manufacturer’s software (HEYEX; Heidelberg Engineering, Heidelberg, Germany). The minimally allowed number of remaining images of a series was nine and only the series with the best image quality was included. The device was regularly calibrated during the study. Color-fundus photography (CFP, 440–650 nm, 60°x55°, image resolution 3680 × 3288 pixel) images were obtained using the Eidon TrueColor Confocal Scanner, CenterVue, Padua, Italy.

**Fig. 1:**
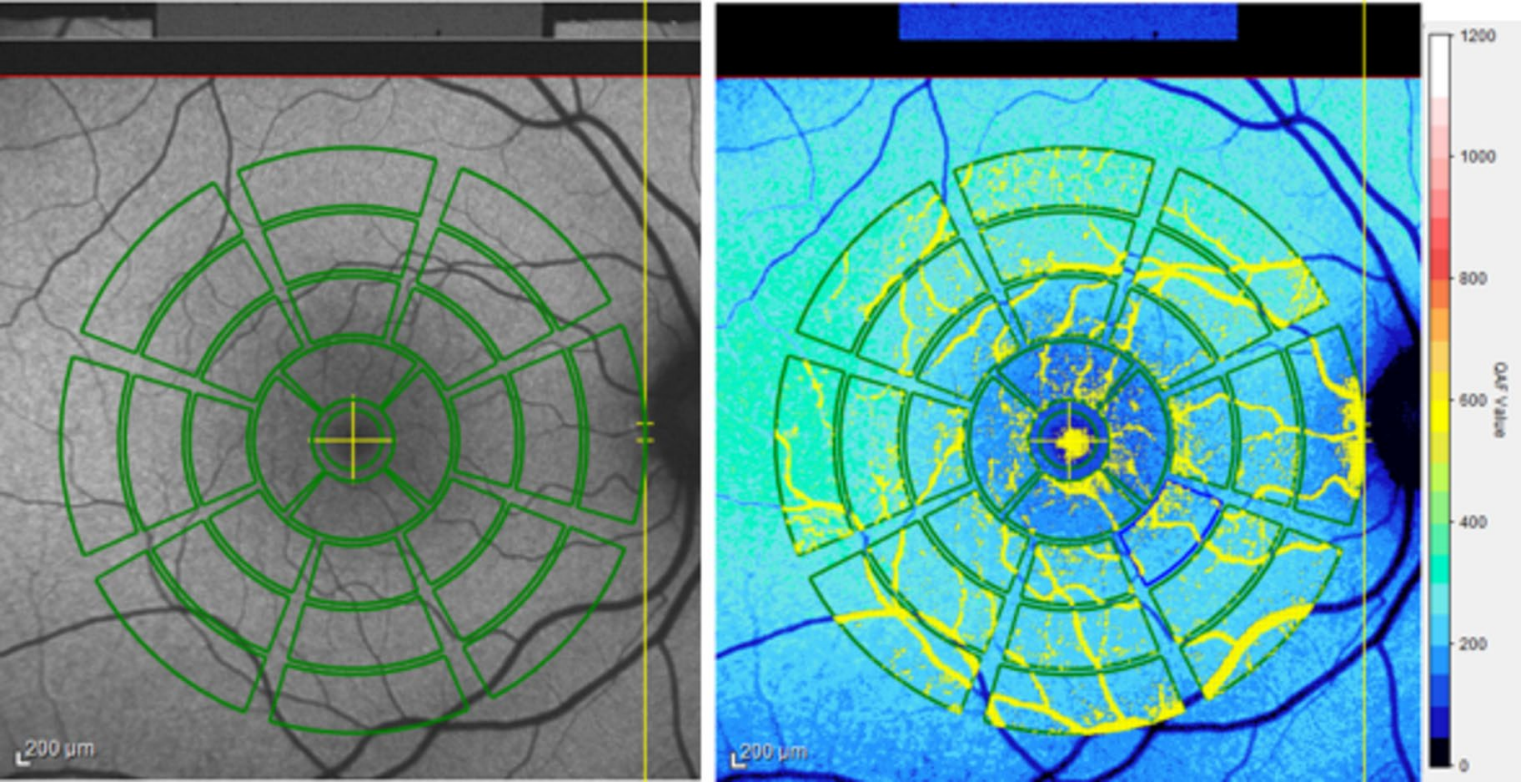
Illustration of quantitative autofluorescence (qAF) image (right eye). Exemplary image of a patient with a clinically unaffected eye (Group 2). Left: grey-scaled qAF view. Right: corresponding color-coded qAF with qAF color-code scale in qAF arbitrary units ([qAF a.u.]). Vessels (yellow) and atrophic retinal lesions (not present in this unilateral unaffected eye) were excluded based on histogram analysis.

**Fig. 2:**
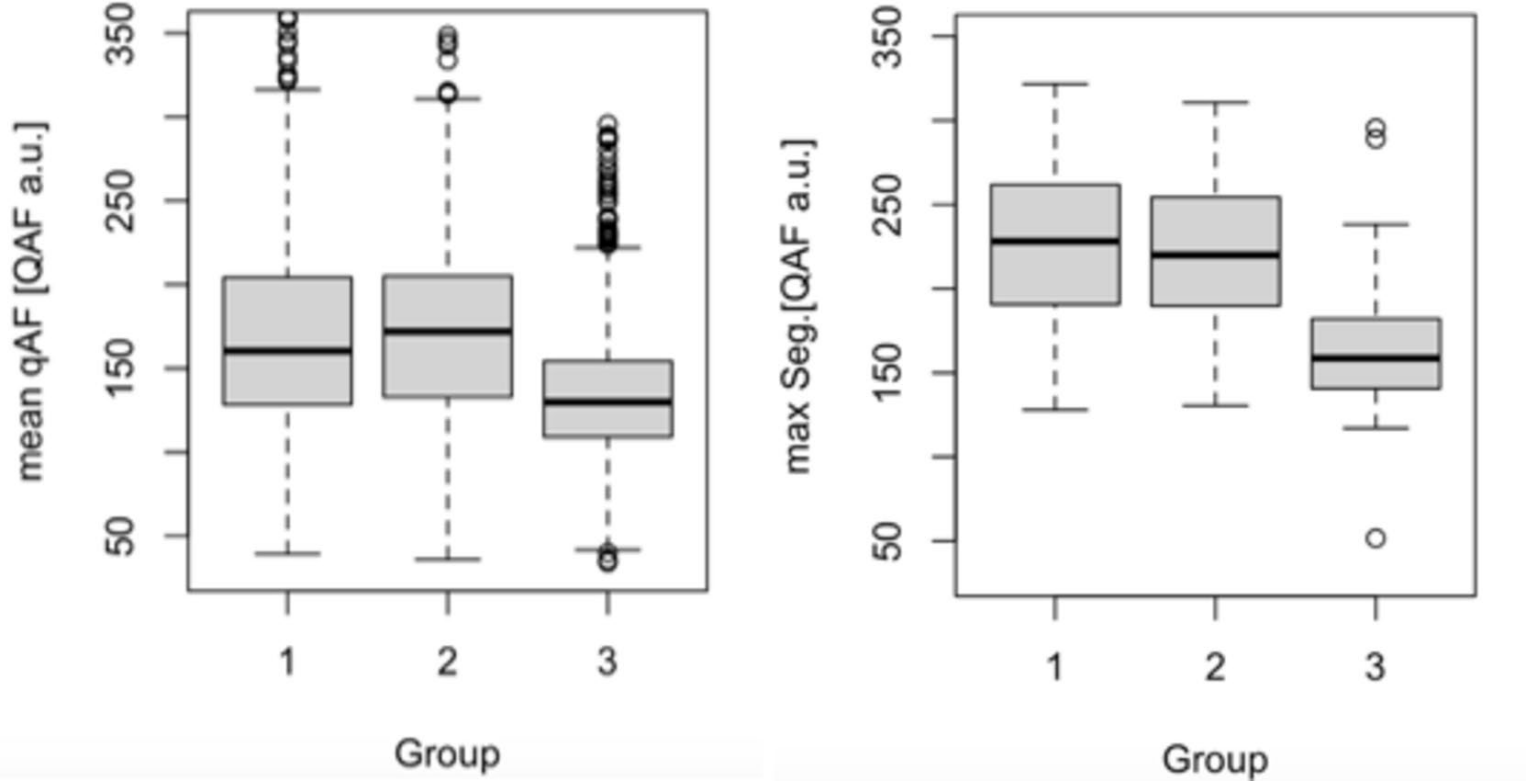
Left: Quantitative autofluorescence (qAF) in posterior uveitis patients. qAF values (in [qAF a.u.]) were significantly higher in clinically affected eyes (Group 1) (p-value between group 1&3:<0.0001) and clinically unaffected eyes (Group 2) (p-value between group 2&3:<0.0001) than in healthy control eyes (Group 3). Right: The segment with the highest qAF value (in [qAF a.u.]) in each patient were significantly higher in clinically affected eyes (Group 1) and clinically unaffected eyes (Group 2) compared to healthy control eyes (Group 3) (p-values:<0.01).

**Fig. 3:**
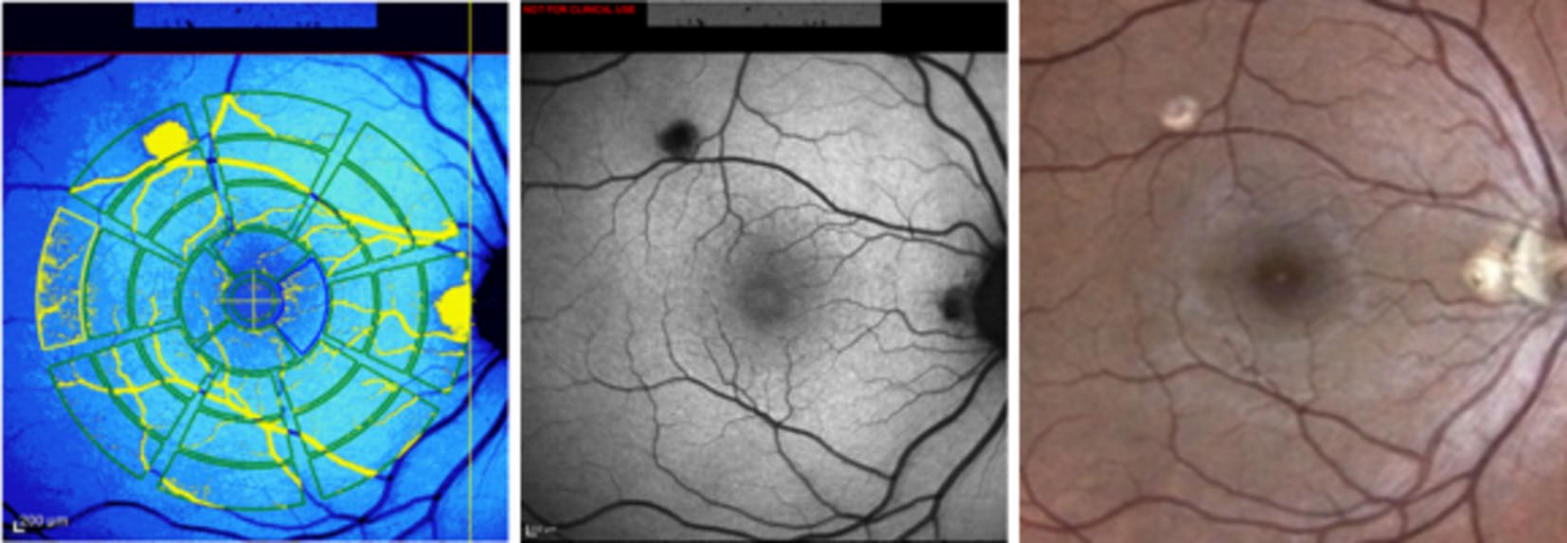
Exemplary images of a female patient with punctate inner choroidopathy (right eye). All images were taken on the same examination date. Left: Color-coded quantitative autofluorescence (qAF) image. Retinal vessels and atrophic lesions were excluded based on histogram analysis by the internal HEYEX software. Middle: corresponding qAF image. Right: color fundus photography.

Images were analyzed and the qAF values were calculated by the system’s inbuilt HEYEX software (Heidelberg Engineering, Figs. 1, 2 and 3) based on Deloris formula^18^. Retinal vessels and atrophic lesions were automatically excluded based on histogram analysis by the internal HEYEX software (Figs. 1 and 3). QAF values at the fovea, the four-segment inner ring, the eight-segment inner ring, the eight-segment middle ring and the eight-segment outer ring were measured in the same manner. Subsequently, the mean qAF value was extracted by averaging the mean of all 29 segments of the qAF grid. (Fig. 1) Additionally, the segment with the highest mean qAF value for each patient was identified and compared across the three groups. QAF values are specified in qAF arbitrary units ([qAF a.u.])^18^.

The qAF measures were compared among three groups. Group 1 comprises patients in whom both eyes were affected by posterior uveitis; however, to minimize potential statistical confounding, only one eye per patient was included in the study. Group 2 consists of patients with one clinically affected and one clinically unaffected eye. In this group, we included only the clinically unaffected eye in the analysis. Group 3 includes healthy, age-matched controls.

### Statistical analysis

Data collection and organization were performed using Microsoft Excel. The statistical analysis was performed with R, version 4.0.2 (R Core Team, Vienna, Austria). *P* < 0.05 was considered statistically significant. The Kruskal-Wallis test was applied to test the null hypothesis that there is no significant difference in mean qAF between the groups. The pairwise Wilcox test was used for post hoc analysis and the Bonferroni method for adjustment. The paired t-test was used to test for difference between the two affected eyes in the same patient. To assess differences in mean qAF values between the three most common entities, we performed an ANOVA to test the null hypothesis of no significant difference.

## Results

### Characteristics of the sample

A total of 83 eyes from 83 subjects were included: 33 affected eyes (33 patients with posterior uveitis; group 1), 21 clinically unaffected eyes (21 patients with unilateral posterior uveitis; group 2), and 29 healthy, age-matched control eyes (29 subjects; group 3). Only one eye per subject was included and every subject was included in only one group. Characteristics of the sample are reported in (Table 1), while the etiologies are detailed in Supplement Table 1.

**Table 1:** Characteristics of the sample.

	Affected eyes in patients with uveitis (group 1)	Unaffected eyes in patients with uveitis (group 2)	Control eyes (group 3)
Eyes (n)	33	21	29
Patients (n)	33	21	29
Age (mean ± SD)	53.7 ± 17.9	43.3 ± 13.0	43.2 ± 19.7
Age (range)	24.1–86.9	20.1–65.5	21.1–75.6
Sex male (n / %)	13 (39.4%)	7 (33.3%)	11 (37.9%)

*SD *  standard deviation, *n*   total number, age in years.

### Comparison of qAF between groups 1–3

All chorioretinal lesions in the clinically affected eyes were inactive at the time of examination. The mean qAF in the clinically affected eyes was 177.0 ± 83.8 [qAF a.u.], while it was 173.8 ± 56.4 [qAF a.u.] in the (yet) clinically unaffected eyes and 135.7 ± 41.8 [qAF a.u.] in the healthy control eyes (Fig. 2, left side). The mean of the segment with the maximal qAF value was 243.2 ± 103.1 [qAF a.u.] in group 1, 227.1 ± 63.4 [qAF a.u.] in group 2 and 168.9 ± 48.5 [qAF a.u.] in group 3 (Fig. 2, right side). The Kruskal-Wallis test revealed significant differences between the posterior uveitis eyes and the healthy control eyes. The post hoc analysis (Pairwise Wilcox test, Bonferroni adjustment method) showed that in both affected and unaffected eyes from patients with posterior uveitis (Group 1 and 2) mean qAF values were significantly higher compared to the age-matched controls (Fig. 2, p-value < 0.0001). No significant difference was observed between the clinically affected eyes (Group 1) and the clinically unaffected eyes (Group 2, Fig. 2, p-value: 0.19). The comparison of the mean of the segment with the highest qAF value yielded similar results (Fig. 2, p-value between Group 1 and 2: 1.0, p-value between group 1 and 3 < 0.001, p-value between group 2 and 3: <0.01). The segment with the highest qAF value was mostly located in the superotemporal quadrant in all three groups, similar as described by Greenberg before^27^. 5 clinically unaffected eyes (23.8%) showed normal mean qAF values.

The macular pigment causes shadowing primarily in the foveal segment at 488 nm excitation, which changes qAF values. Therefore, a separate analysis excluding the foveal segment from the analysis was conducted, which, however, did not show significantly different results(Supplement Table 3). To control for a possible confounding effect of mild lens opacities, we analyzed the image quality scores from Heidelberg Engineering OCT images, which were performed on the same day of qAF examination. The overall mean score was 36.5 and there were no statistically significant differences between the groups (p-value = 0.45, Group 1: 35.1 ± 3.9, Group 2: 34.8 ± 4.9, Group 3: 36.5 ± 4.4, data unavailable for one patient in group 1).

In all three groups, qAF values were statistically significantly (p-value < 0.05) higher in females than in males (Supplement Table 2), as previously described in healthy eyes by Greenberg and colleagues^27^.

### Comparison of qAF between affected and unaffected eyes within the same patients

We were able to compare 10 out of 21 eyes, since in some patients the image quality of the contralateral eye was insufficient or the lesions were too large to analyze the background qAF. Both eyes were imaged on the same date. The mean qAF value in the unaffected eye was 182.8 ± 43.1 [qAF a.u.] and 177.0 ± 36.6 [qAF a.u.] in the clinically affected eye in the same patient. There was no significant difference (p-value 0.68) between the two eyes.

### Comparison of qAF between different uveitis etiologies

Due to limited sample size, we restricted our comparison to the three most frequent diagnoses. There was no significant difference (p-value = 0.96) in mean qAF values between the three most frequent diagnoses (idiopathic panuveitis, sarcoidosis, and APMPPE).

## Discussion

Our study revealed increased qAF values in both clinically affected and unaffected eyes in patients with posterior uveitis compared to healthy, age-matched controls. There was no significant difference in autofluorescence values between the clinically affected and (yet) clinically unaffected eyes. Our results may indicate some degree of low-grade inflammation / metabolic alteration visible on qAF outside the visible lesions in inactive posterior uveitis which might also be present in the clinically unaffected fellow eye.

To the best of our knowledge, this study is the first to assess background autofluorescence outside chorioretinal lesions in both affected and unaffected eyes in posterior uveitis as well as controls. FAF is an established and reliable measure of RPE function and integrity, as well as aids in identifying and grading chorioretinal lesions and RPE damage^16,21^. The autofluorescence observed on FAF imaging primarily originates from fluorophores, such as bisretinoids and other currently unknown substances, present in both photoreceptors and RPE cells^10,13^. Bisretinoids form through non-enzymatic reactions as by-products of the visual cycle and accumulate in the lysosomes of RPE cells as a major component of lipofuscin (LF) and melanolipofuscin^28–31^. As stated in previous studies, cell impairment and degeneration, as also seen in posterior uveitis, may result in excessive production of bisretinoid fluorophores in photoreceptor cells^13,32,33^. Additionally, the impaired RPE cells may have reduced capacity to process and clear bisretinoids, leading to their accumulation and consequently an increase in autofluorescence.

The increased qAF levels in posterior uveitis patients could also be caused by increased metabolic activity in the RPE cells and photoreceptors, as previously discussed^21,34,35^. Earlier research has indicated a higher production of substances with potential autofluorescence characteristics, utilizing alternative metabolic pathways^22,36–38^. Inflammatory reactions, as observed in posterior uveitis, increase the metabolic activity through the release of inflammatory mediators and the activation of immune cells^39,40^. This may have toxic effects especially on the outer segment of the photoreceptors through the increased generation of reactive oxygen species (ROS) by immune cells. The outer segments are prone to damage through ROS due to their high content of Docosahexaenoic acid (22:6), a polyunsaturated fatty acid which is especially susceptible to in vivo peroxidation^41–43^. This process leads to a disarray and disorganization of discs and cellular membranes, ultimately resulting in the shortening or loss of the outer photoreceptor segments. This may result in a relatively increased autofluorescence, attributed to a window defect caused by photopigment loss and a loss of the outer segment^41,44,45^. The increased qAF values observed in clinically affected and unaffected eyes may be attributed to impaired photoreceptor and RPE cells and some degree of photopigment loss due to a low grade inflammation and the subsequently increased production and accumulation of fluorophores.

A previous study in patients with uveitis revealed elevated cytokine and chemokine tear levels in both active and inactive uveitis eyes compared to healthy controls^46^. Similar to our study, Carreño et al. showed no difference in the observed inflammatory biomarker between active and inactive uveitis eyes when compared to their contralateral eyes. However, the comparison of this study with ours is limited due to the different analyzed biomarker (cytokine/chemokine vs. qAF), the inclusions of any uveitis type in our study, and the less elevated levels in posterior uveitis.

Previous cell culture studies have shown that LF destabilizes the lysosomes of the RPE cells and triggers the secretion of inflammatory cytokines as well as the activation of the inflammasome^47^. Additionally, Zhou and colleagues showed that the photooxidation of LF components could activate the complement system^20,48^. However, histological studies using human donor eyes, revealed an increase in LF load during aging but could not show any RPE cell loss despite LF abundance - a finding that questions direct LF toxicity but cannot rule out that individual fluorophores have the potential of harm^49^. In addition, increased qAF intensity levels have recently been shown in in vivo studies examining patients with systemic rheumatic diseases and chloroquine/hydroxychloroquine intake. Interestingly, these patients also showed an overall increase of qAF levels throughout the posterior pole as compare to healthy controls, a similar finding as in the current study^35,50^. Therefore, we suggest that systemic diseases might impact the metabolism of bisretinoids (the major contributors to FAF) leading to new or altered autofluorescent products.

The RPE cells actively support immune privilege by regulating T-cell proliferation and INF-y production through expression of soluble and surface molecules (TGF-ß, PEDF, CD46, CD59, CD95)^51–53^. As previously discussed in the context of sympathetic ophthalmia, inflammatory neuropeptides, the activation of the inflammasome and the complement system, may also induce a (subclinical) inflammatory reaction in the yet unaffected eye^9^. Our results of an increased autofluorescence signal in both clinically affected and unaffected eyes in patients with posterior uveitis may indicate that the underlying mechanisms in posterior uveitis partially abrogate immune privilege in both the affected and clinically unaffected eye as shown in retinal laser burn and corneal surgery before^54,55^. The abrogation of immune privilege may lead to a subclinical inflammatory response in the unaffected eye, potentially increasing the risk of future disease.

Strengths of this study include the prospective study design, the availability of a quantitative measure for FAF and the standardized phenotyping of posterior uveitis based on SUN criteria. Additionally, the study benefits from the comparison to age-matched controls as well as the relatively large total sample size for posterior uveitis. The limitations of our study are its cross-sectional nature, the heterogeneity of diagnoses among patients, that we did only include inactive disease, and the known limitations of qAF, including inter-examiner reliability issues^12^. Due to the cross-sectional nature of the study, we cannot assess possible aftereffects and changes in qAF values over the course of the inactive phase. QAF may increase or decrease after active inflammation due to effects such as further photoreceptor damage or RPE loss. We chose to include only inactive uveitis eyes in this study, as it is much more challenging to include a sufficient number of eyes with active disease due to the relatively low prevalence of active posterior uveitis eligible for inclusion in such a study. Further, eyes with active disease would have to be analyzed in additional separate groups, as the active inflammation could otherwise confound the analysis. Variations in disease courses, such as differences in treatment, relapse frequencies, and time elapsed since the last active phase, may have influenced qAF values in clinically affected eyes. Longitudinal studies with larger sample sizes, including both active and inactive stages, are needed to accurately evaluate differences in qAF values throughout disease stages and their potential prognostic value. We minimized the potential confounding effect of the lens on qAF imaging by excluding patients with relevant cataract; however future studies could use new metrics and, therefore, also account for lens opacities^56,57^. Additionally, we further minimized potential influences of mild lens opacities by analyzing the image quality scores from Heidelberg Engineering OCT images, and found no significant differences between the groups with a good overall score. Excluding retinal vessels and lesions through histographic analysis results in the exclusion of the fovea in some patients. As shown in the results, this effect is most likely not relevant. The rarity of all forms of posterior uveitis complicates the analysis of a large number of cases, especially when applying special examination techniques like qAF.

In conclusion, we found significantly increased average qAF values in both clinically affected and unaffected eyes of patients with inactive posterior uveitis. These results might indicate low grade inflammation possibly related to increased metabolic activity due to some degree of subclinical inflammation in currently inactive and clinically (yet) unaffected eyes. Our results suggest that the mechanisms leading to increased background FAF affect both the clinically affected and unaffected eyes to a similar extent. QAF may serve as a quantitative imaging endpoint in posterior uveitis patients and may be able to detect inflammation before it becomes clinically apparent. Further research and longitudinal studies also including active disease are warranted to better understand the underlying mechanisms and whether these changes are associated with future clinically visible inflammation, and therefore allow earlier detection and treatment options to prevent irreversible damage.

## Electronic supplementary material

Below is the link to the electronic supplementary material.

Supplementary Material 1

## Data Availability

Original data is available from the corresponding author upon reasonable request.
