# Supplementary material for: Quantitative autofluorescence is increased in clinically unaffected fellow eyes from patients with posterior uveitis
Source: Sci Rep. 2025 Feb 26;15:6952. doi: 10.1038/s41598-025-90071-7 (PMC11865583; doi:10.1038/s41598-025-90071-7)
Supplement: Supplementary file 1 — Supplementary Material 1 [file 41598_2025_90071_MOESM1_ESM.docx]

**Supplement Table 1.** Uveitis diagnosis in group 1 and 2.

|  | Group 1 (affected) | Group 2 (unaffected) |
| --- | --- | --- |
| Idiopathic posterior uveitis | 2 (6.1%) | 5 (23.8%) |
| Idiopathic panuveitis | 9 (27.3%) | 1 (4.8%) |
| Birdshot chorioretinopathy | 2 (6.1%) | 2 (9.5%) |
| Sarcoidosis | 5 (15.2%) | 2 (9.5%) |
| Acute posterior multifocal placoid pigment epitheliopathy (APMPPE) | 6 (18.2%) | 1 (4.8%) |
| Acute retinal pigment epitheliitis | 1 (3.0%) | 0 (0%) |
| Punctuate inner chorioretinopathy (PIC) | 3 (9.1%) | 1 (4.8%) |
| Multifocal choroiditis and panuveitis (MCP) | 1 (3.0%) | 3 (14.3%) |
| Neuroretinitis | 1 (3.0%) | 1 (4.8%) |
| Multiple evanescent white dot syndrome (MEWDS) | 0 (0%) | 2 (9.5%) |
| Acute zonal occult outer retinopathy | 1 (3.0%) | 2 (9.5%) |
| Behcet’s Disease | 2 (6.1%) | 0 (0%) |
| Cytomegalovirus retinitis | 0 (0%) | 1 (4.8%) |

**Supplement Table 2.** Mean quantitative autofluorescence (qAF) in qAF arbitrary units and standard deviation in females and males.

|  | Uveitis | Unaffected | Control |
| --- | --- | --- | --- |
| Female | 190.73 ± 99.7 | 182.1 ± 58.4 | 140.3 ± 44.9 |
| Male | 157.4 ± 46.7 | 157.3 ± 48.0 | 128.0 ± 34.9 |

**Supplement Table 3.** Quantitative autofluorescence (qAF) results in qAF arbitrary units with and without fovea segment

|  | Uveitis | Unaffected | Control |
| --- | --- | --- | --- |
| Mean with fovea | 177.0 ± 83.8 | 173.8 ± 56.4 | 135.7 ± 41.8 |
| Mean without fovea | 180.1 ± 83.1 | 177.0 ± 54.4 | 138.0 ± 40.2 |
